# Influence of periodontal conditions on quality of life in patients with chronic kidney disease: A systematic review and meta-analysis

**DOI:** 10.4317/medoral.28048

**Published:** 2026-04-19

**Authors:** Caroline Schöffer, Catiusse Crestani Del'Agnese, Emanuelle Ledermann Marder, Leandro Machado Oliveira, Raquel Pippi Antoniazzi

**Affiliations:** 1 Postgraduate Program in Dentistry, Periodontics, Department of Stomatology, Federal University of Santa Maria, Santa Maria, Brazil; 2 Undergraduate in Dentistry, Federal University of Santa Maria, Santa Maria, Brazil

**Keywords:** Kidney diseases, periodontal diseases, periodontitis, quality of life, oral health-related quality of life.

## Abstract

**Background:**

Chronic kidney disease (CKD) is associated with a higher occurrence of periodontitis, which may be linked to negative psychosocial and physical impacts on the lives of patients affected by both conditions. Therefore, the aim of this systematic review was to evaluate whether periodontal conditions influence OHRQoL in patients with CKD.

**Materials and Methods:**

Electronic searches were performed in five databases up to May 2025. Observational studies conducted in individuals with CKD that investigated the relationship between periodontal conditions through clinical examinations and OHRQoL, were included. Meta-analyses were performed to estimate the mean difference (MD) in OHRQoL, as well as the correlation.

**Results:**

Out of the 2036 potentially eligible reports, 7 studies of patients with end-stage renal disease (ESRD) were included. Pooled estimates demonstrated that OHIP-14 scores are higher in individuals with ESRD compared to healthy controls. Impaired OHRQoL can be attributed to the higher prevalence of periodontitis in renal patients. Considering a score-response gradient, those with stage III/IV periodontitis had higher OHIP-14 scores compared to patients without periodontitis or with stage I/II periodontitis (MD:1.88; 95%CI: 0.67-3.09; p=0.002). Pooled estimates demonstrated a weak but significant positive correlation between periodontal probing depth (r=0.145; p=0.028) and plaque index (r=0.385; p<0.001) with OHIP-14 scores.

**Conclusions:**

Severe periodontitis, along with higher probing depth and plaque scores, negatively influences the OHRQoL of patients with ESRD. However, these relationships show a small magnitude.

**Introduction** 

Chronic Kidney Disease (CKD) is a long-term condition characterized by an irreversible damage that occurs in the kidney structure, thereby impairing kidney function. Evidence of renal damage is identified by the reduction in filtration capacity of the renal functional units (nephrons), measured by the reduction in glomerular filtration rate (GFR). CKD is categorized into five stages, being the last (end-stage) when kidney failure occurs, requiring the patient to undergo renal replacement therapy or transplantation. This diagnosis is established from a GFR lower than 15 mL/min per 1.73 m² [1]. It is estimated that around 10% of the global population currently have CKD [2]. The prevalence of CKD is increasing, generating high costs to the public health systems [3]. It is estimated that about 1.2 million people lost their lives due to kidney failure in 2015, with an increase of 32% in 10 years [4]. Patients affected by CKD tend to have a progressive worsening of their health-related quality of life as the disease progresses [5], mainly related to physical health decline [6].

Periodontitis is characterized by microbially associated, host-mediated inflammation, which clinically results in loss of periodontal attachment, periodontal pockets, and bleeding [7]. Such a disease represents a global public health problem, with the age-standardized prevalence of severe periodontitis reaching 12.50% [8]. The relationship between periodontitis and systemic diseases has been extensively studied, demonstrating that the local inflammatory response and infection triggered by periodontitis have systemic implications [9]. An umbrella review described a bidirectional relationship between periodontitis and CKD, emphasizing that these individuals exhibit greater attachment loss and probing pocket depth (PPD), higher amounts of dental plaque and calculus compared to systemically healthy individuals [10]. Additionally, non-surgical periodontal treatment reduces systemic inflammatory burden and can improve renal function [10]. Therefore, individuals with CKD are at increased risk of periodontitis, and periodontal treatment may contribute to the systemic health of these individuals.

Patients with periodontitis can experience swollen and retracted gums, halitosis, tooth mobility, as well as tooth loss, chewing dysfunction, and aesthetic impairment that affect the psychological, social, and physical aspects of their lives [11]. Consequently, periodontitis impairs the oral health-related quality of life (OHRQoL), as observed in several systematic reviews, with the negative impact worsening with the severity of periodontitis [12-15]. However, these reviews have seldom explored this association in specific populations, such as individuals with CKD.

Considering the bidirectional association between CKD and periodontitis, their shared risk factors, and the high worldwide prevalence of severe forms of these diseases, their unfavorable psychosocial and physical effects on OHRQoL may accumulate. Therefore, the aim of this systematic review was to investigate the association between periodontitis and impaired OHRQoL in individuals with CKD. Furthermore, it aimed to correlate periodontal parameters with OHRQoL scores in this population.

## Materials and Methods

This systematic review is reported according to the PRISMA (Preferred Reporting Items for Systematic Review and Meta-Analyses) statement [16]. PRISMA 2020 checklist is presented in Supplementary 1 (https://www.medicinaoral.com/medoralfree01/28048/Suppl1.pdf). It was registered on PROSPERO (number CRD42024547256) before commencing the review process.

*Research question and eligibility criteria*

This systematic review aimed to answer the following focus questions (FQ) according to the PECOS acronym: Question 1 (FQ1): Do adult individuals (≥18 years old) with CKD (Population) and periodontitis or severe periodontitis, as defined by the authors through clinical periodontal examination (clinical attachment loss (CAL) and/or PPD); (Exposure), experience a greater negative impact on OHRQoL, measured using validated instruments (Outcome), compared to those without periodontitis or with initial or moderate forms of the disease (Comparator)?  Question 2 (FQ2): Are clinical periodontal parameters (exposure) and OHRQoL scores (outcome) correlated in adults with CKD (Population)?

Observational studies (longitudinal, case-control, or cross-sectional) were included without restrictions on language or publication date. Studies involving populations with acute kidney disease, those with periodontal conditions assessed through self-report, those assessing only general quality of life, and those that did not report data on the relationship between periodontal conditions and OHRQoL were excluded.

*Search strategy and data extraction*

Searches were conducted in MEDLINE (via PubMed), EMBASE, LILACS, Web of Science and Scopus to identify studies relevant to the research question. Grey literature was searched using the DANS database. The searches were performed up to May 2025, and the search strategies are presented in Supplementary 2 (https://www.medicinaoral.com/medoralfree01/28048/Suppl2.pdf). 

Duplicate studies were removed using the Rayyan tool (https://www.rayyan.ai/). To avoid duplicate selection of studies, comparisons were made based on authors' names/affiliations and study characteristics. In cases of uncertainty, up to three weekly attempts were made to contact the corresponding authors for clarifications.

Reviewers were trained on the eligibility criteria before commencing the study. Two reviewers (C.S. and C.C.D.) individually selected studies in duplicate. Titles and abstracts were initially analyzed, and studies that met the eligibility criteria or provided insufficient information about our research question were selected for full-text appraisal. Reasons for excluding studies at this stage were documented Supplementary 3 (https://www.medicinaoral.com/medoralfree01/28048/Suppl3.pdf). In cases of disagreement between the reviewers, decisions were resolved through consensus; if consensus could not be reached, a third reviewer (R.P.A.) made the final decision. 

The two same reviewers (C.S. and C.C.D.) independently extracted the following data from the included studies in duplicate: Authors, year of publication, country, sample and design; details of CKD, periodontal examinations, diagnosis and prevalence of periodontitis; OHRQoL assessment instrument; adjustment for confounders; OHRQoL scores, correlation between periodontal clinical variables and OHRQoL, influence of periodontal conditions on OHRQoL and domains affected.

*Quality assessment*

Quality assessment of the included studies was conducted by two reviewers independently (C.S. and C.C.D.), using the Newcastle-Ottawa scale [17] adapted for cross-sectional studies [18,19]. Supplementary 4 (https://www.medicinaoral.com/medoralfree01/28048/Suppl4.pdf). Disagreement between reviewers was resolved by open discussion. If agreement was not reached, the final decision was made by another co-author (R.P.A.). For the purposes of our research question, the case*-*control study was considered equivalent to a cross-sectional study with a control group. This adapted scale comprises seven items distributed across four dimensions: Selection; comparability; exposure and outcome. Two domains, selection and adjustment for confounders (in the outcome dimension), can each receive two points. Therefore, the total score obtained by each study ranges from 0 to 9 points [17]. Scores of 0*-*3 were considered indicative of low quality, 4*-*6 points indicated moderate quality, and ≥7 points were considered indicative of high-quality studies [19].

*Certainty of evidence*

The GRADE pro tool was used to determine the certainty of the evidence [20]. Two examiners (C.S. and C.C.D.) determined the overall strength of the evidence. A summary of the results found is presented in Supplementary 5 (https://www.medicinaoral.com/medoralfree01/28048/Suppl5.pdf).

*Statistical analysis*

The analyses were conducted using the “meta” and “metafor” packages of R Statistical software (version 4.3.3). Exploratory meta-analyses with random effects model were used to pool periodontitis prevalence and OHIP-14 scores (mean and 95% confidence intervals) when comparing individuals with CKD and healthy individuals. Another meta-analysis combined mean differences (MD) in OHIP-14 scores between individuals with severe periodontitis and those without periodontitis or with initial or moderate periodontitis (FQ1). Moreover, pooled correlation coefficients of plaque index and PPD with OHRQoL scores were calculated using Fisher's r-z transformation (z) in a random effects model (FQ2). Heterogeneity between studies was assessed by Cochran’s Q test and I^2^ statistics, ranging from 0 to 100%, to quantify inconsistencies between studies. I2<25%, 25% to 75% and ≥75% represent low, medium and high heterogeneity, respectively [21]. The 95% prediction interval (PI) was also calculated. 

## Results

A total of 2036 studies were retrieved from the databases, out of which 19 were selected for full-text appraisal. Among these, seven studies met the eligibility criteria and were included in the present study. A flowchart is shown in Figure 1. The excluded studies after full-text reading and the reasons for their exclusion can be found in Supplementary 3 (https://www.medicinaoral.com/medoralfree01/28048/Suppl3.pdf).

The characteristics and main results of the included studies can be found in Table 1. Six studies used a cross-sectional design, while one was a case-control study, and they were conducted across five different countries. All studies included patients with end-stage renal disease (ESRD), recruited from nephrology clinics who were on regular hemodialysis therapy. One study also included a group of patients after kidney transplantation [22]. The number of participants ranged from 47 to 767. Regarding the assessment of periodontal conditions, only two studies conducted full-mouth examinations [22,23], three conducted partial examinations, and two did not report examination details. Two studies [22,23] defined periodontitis according to the criteria proposed by Eke *et al*. [24] and Page and Eke [25], respectively. Two studies assessed the periodontal status using the Community Periodontal Index (CPI) [26,27] and another employed the Periodontal Disease Index Score (PDI) [28]. The remaining two studies presented data on the evaluated periodontal parameters without defining periodontitis [29,30].

Regarding OHRQoL instruments, four studies used only the OHIP-14, and three used both the OHIP-14 and the GOHAI. The OHIP-14 was interpreted such that higher scores indicated worse OHRQoL (range: 0*-*56). Two studies applied additive (ADD; summed continuous scores) and simple count (SC; binary outcome) scoring methods for both the OHIP-14 and the GOHAI [28,29]. In both studies, GOHAI scores were interpreted similarly to the OHIP-14, with higher scores indicating worse OHRQoL [28,29].

The association between periodontitis and OHRQoL was explored in two studies [22,23]. One study reported that individuals with mild to moderate periodontitis had worse OHRQoL compared to those without periodontitis (RR=1.49; 95% CI 1.16-1.91) and this impact was even greater for individuals with severe periodontitis (RR=1.77; 95% CI 1.36-2.30) [23]. Pakpour *et al*. [26] reported a significant association between CPI scores and OHRQoL (*p<*0.05). Hajian-Tilak *et al*. [28] and Schmalz *et al*. [30] reported a significant correlation between OHRQoL scores and both PDI and PPD, respectively. Two studies reported a positive correlation between plaque index and OHIP-14 scores [28,29]. Two other studies did not show significant associations between periodontal status and OHRQoL [22,27].

Two studies adjusted for confounding factors [23,26], including demographic, socioeconomic, behavioral, general health, general quality of life, and oral health variables. Only one study assessed the OHIP-14 domains, reporting that physical pain, physical disability, psychological disability, and psychological discomfort were affected by periodontitis [23].

*Methodological quality*

The quality assessment of the included studies is presented in Table 2. Two studies showed a moderate quality [23,26], while five studies were categorized as having low quality [22,27-30]. Only one study [30] had a truly representative sample selection. Furthermore, two studies had calibrated examiners for exposure measurements [23,26].  Regarding comparability, four studies included appropriate comparison groups [23,26,27,30]. Most studies had limitations concerning the outcome dimension, particularly due to the lack of adjustment for confounders and the failure to report the response rate.

*Meta-analysis*

All seven studies presented mean OHIP-14 scores in individuals with ESRD. However, one study was not included in the analysis because it used a different scoring scale (0*-*70) [27], whereas the remaining studies applied the proposed 0*-*56 range. The exploratory meta-analysis showed that OHIP-14 scores were higher in individuals with ESRD compared to systemically healthy individuals (12.29 vs. 5.59, n=6 studies) (Figure 2). The impaired OHRQoL in patients with ESRD might be partially attributed to the higher prevalence of periodontitis observed in these individuals (84.2% vs. 59.7%, n= 3 studies) (Figure 3). 

Figure 4 (FQ1) presents the mean differences (MD) in OHRQoL scores (OHIP-14), estimated at 1.88 (95% CI: 0.67*-*3.09, *p=*0.002). The pooled data from two studies indicated that individuals with stage III/IV periodontitis experience a more pronounced negative impact on OHRQoL compared to those without or with stage I/II periodontitis. The observed values range from 0.67 [22] to 8.50 [23]. It should be noted that there was low heterogeneity observed (I2=11.5%), and the certainty of the evidence was very low in Supplementary 5 (https://www.medicinaoral.com/medoralfree01/28048/Suppl5.pdf).

Correlation meta-analyses were conducted using data from three studies (Table 3; FQ2). A weak positive correlation (r=0.145; *p=*0.028) was observed between PPD and OHIP-14 scores [29,30]. Another meta-analysis showed a moderate positive correlation (r=0.385; *p<*0.001) between plaque index and OHIP-14 scores [28,29]. The studies were quite homogeneous (I2=0%) and the certainty of the evidence was very low in Supplementary 5 (https://www.medicinaoral.com/medoralfree01/28048/Suppl5.pdf). Meta-analysis using the GOHAI instrument yielded similar results for plaque index.

## Discussion

To our knowledge, this is the first systematic review to evaluate the impact of periodontal conditions on OHRQoL of patients with CKD, estimating pooled mean scores and correlation coefficients. Our findings indicate that periodontitis impairs the OHRQoL of patients with ESRD. Furthermore, inflammatory parameters and dental biofilm showed correlations with OHRQoL scores. However, the strength of these relationships is small and highly variable.

Periodontitis has a negative impact on OHRQoL [12-15], due to functional, aesthetic, and social consequences [11]. Systemically healthy individuals with periodontitis experience impairment in OHRQoL, with a higher mean OHIP-14 score compared to controls (mean difference =5.14; 95% CI 3.64-6.64) [15]. The results obtained from our global estimates, despite the very low certainty of evidence, also showed a negative impact of periodontitis on OHRQoL. Severe periodontitis was associated with more impaired OHRQoL, although the difference, compared to individuals without periodontitis or with initial-stage periodontitis, was smaller. Patients with ESRD often have associated comorbidities that can further worsen their perception of quality of life [31]. On the other hand, although periodontitis is more prevalent in individuals with CKD, it may have a lower impact on OHRQoL due to the significant vulnerability of this population, which experiences a greater burden of diseases, with more severe and debilitating outcomes compared to oral diseases. In more debilitating diseases, such as leukemia, rheumatoid arthritis, or in patients after transplantation, the association between periodontitis and impaired OHRQoL was not observed [32-34]. Schmalz *et al*. [35] conducted a systematic review that addressed the impact of oral conditions on OHRQoL in patients undergoing renal replacement therapy, reporting results similar to ours regarding periodontal conditions. Furthermore, associations were found between OHRQoL and diabetes, blood parameters, and duration of dialysis, reinforcing other factors relevant to the well-being of this population. However, in this review [35], no quantitative analysis was conducted regarding the impact of periodontal condition on OHRQoL, making it difficult to interpret the current evidence. 

Pooled estimates showed a positive correlation between probing depth and plaque index with OHIP-14 scores. Various periodontal clinical parameters were associated with OHRQoL in different studies. PPD measurements were the most common used, as they reveal the current inflammatory status of the disease and thereby the complexity of periodontal treatment. Additionally, in the absence of CAL, which represents the cumulative destruction of the support apparatus over a lifetime, PPD can be associated with gingival recession, a significant factor compromising OHRQoL [12]. Advanced periodontitis is related to higher tooth mobility, pain, and exposed root surfaces, leading to embarrassing smile exposure [36] or hypersensitivity. Similarly, dental biofilm is associated with gingival inflammation and is consequently a risk factor for periodontitis [37]. Therefore, it is likely that PPD and plaque contribute to impaired quality of life.

Individuals with CKD are more likely to have periodontitis and consequently more severe CKD. Although the two conditions share several risk factors, their relationship might involve additional causes [9]. The biological plausibility of the systemic effects of CKD on the oral cavity is multifaceted, including factors such as medications used, dialysis status, immunological dysfunction, diseases that may serve as confounding variables, and inadequate oral hygiene. Furthermore, individuals with CKD are immunocompromised due to dysfunctional T and B lymphocytes and PMNs. Thus, the pathogenesis of CKD induces systemic inflammation and creates a favorable oral environment for the colonization and persistence of periodontal pathogens. However, it is worth noting that the relationship between CKD and periodontitis may be syndemic rather than causal, with these conditions coexisting and an increasing inflammatory load worsening each condition [10]. 

The instruments used to evaluate OHRQoL in patients with CKD were OHIP-14 and GOHAI, which tend to present concordant results. Both are widely used in dental research, as they measure the impact of oral health on social, psychological, and physical aspects of individuals' lives. The OHIP-14 is widely endorsed for its brevity, comprehensiveness, simplicity, and established psychometric properties [38]. It is a multidimensional instrument composed of 14 items that assess the impact in seven different dimensions [39], similar to GOHAI, which has 12 items and assesses three different dimensions. 

The present review has limitations, primarily due to the number and heterogeneity of included studies, which stem from the varied methods for the assessment of periodontal conditions and the lack of analyses adjusted for possible confounding factors. Most primary studies reported results from bivariate analyses, such as mean differences between groups and correlations between periodontal parameters and OHRQoL. Only two studies presented associations through multivariate analysis, reducing the risk of over or underestimating the reported impacts of periodontal conditions on OHRQoL [23,26]. Furthermore, all studies exhibited a moderate to low quality, diminishing the reliability of the reported estimates. The limited number of studies also underscores the necessity for cautious interpretation of our results, as only few studies provided data suitable for compiling global estimates, thus precluding large global samples. It should be noted that these findings can only be generalized to patients with ESRD. 

## Conclusions

Periodontitis appears to negatively impact OHRQoL in individuals with end-stage renal disease. Moreover, OHRQoL impairment was positively correlated with increased probing depth and plaque scores. However, it is important to emphasize that the magnitude of this association was small, and the existing evidence remains scarce, heterogeneous, and of very low methodological quality. Further well-designed studies are needed to clarify these relationships and confirm the observed effect size.

## Supplementary Material

Supplementary 1

Supplementary 2

Supplementary 3

Supplementary 4

## Figures and Tables

**Table 1:** Table 1: Characteristics of the studies included in this systematic review (n=7).

**Author, ** **Year ** **Country**	**Design** **Kidney disease and control**	**Sample size (n) Gender (%) Age (years) ** *(Mean±SD) or percentage (%)*	**Clinical periodontal examinations Periodontal diagnosis Periodontitis prevalence** **n (%)**	**OHRQoL instrument Adjusted for confounders**	**Association (A): ** *Exposure versus (vs) Comparison* ** Correlation (C): ** *Periodontal clinical variables and OHRQoL scores*
Oliveira *et al.* 2020 [23] Brazil	Cross-sectional Hemodialysis patients: Dialysis centers Control: None	n:180 55.0% Male 51.98±14.34	Visible plaque index, gingival bleeding index, PPD, CAL and BOP Eke *et al*. 2012 [24]: Absent, mild, moderate, and severe periodontitis Prevalence: Mild/moderate: 82 (45.5%) Severe: 74 (41.1%)	OHIP-14 Total scores Domain-specific Skin colour, age, schooling, smoking, visible plaque index	A: Severe periodontitis (Stage III/IV) # vs No periodontitis
Kahar *et al.* 2019 [27] United States	Cross-sectional Hemodialysis patients: Three dialysis centers Control: None	n: 68 60.3% Male ≤65 (44.12%*) >65 (55.88%*)	CPI NR	OHIP-14 GOHAI Total scores NR	A: CPI≥1 vs CPI=0
Schmalz *et al.* 2018 [30] Germany	Cross-sectional Hemodialysis patients: Three dialysis centers Control: None	n: 210 35.0% Female 64.92±15.7	PPD PPD≥4mm NR	OHIP-14 Total scores NR	A: PPD≥4mm vs PPD<4mm and C: PPD and OHIP-14 scores
Schmalz *et al.* 2016 [22] Germany	Cross-sectional Hemodialysis patients: Five university-affiliated dialysis centers Kidney transplantations recipients: Transplant outpatient clinic of the University Medical Center (≥5 years post-transplantation) Control: Healthy individuals: Department of Preventive Dentistry, Periodontology and Cariology of the University Medical Center	Hemodialysis: n:87 37.9% Female 60.98±14.01 Kidney transplantations: n:39 51.3% Female 56.51±11.56 Healthy: n:91 65.9% Female 58.31±9.91	PPD and CAL Page & Eke, 2007 [25]: no/mild, moderate and severe periodontitis Prevalence moderate/ severe periodontitis: Hemodialysis: 84 (96.6%) Kidney transplantations: 34 (87.2%) Healthy: 72 (79.1%)	OHIP-14 Total scores NR	A: Moderate/severe periodontitis (Stage III/IV) # vs No/mild periodontitis (No/Stage I/II) #
Pakpour *et al.* 2015 [26] Iran	Case-control Hemodialysis patients: Four dialysis centers Control: Healthy individuals: Health centers	Hemodialysis: n:512 62.9% Male 57.7±17.01 Healthy: n:255 62.0% Male 55.8±15.9	Gingival index (GI), modified Quigley-Hein index visual plaque index (QVPI) and CPI Prevalence: Hemodialysis: CPI 3: 162 (31.6%) CPI 4: 74 (14.5%) Healthy: CPI 3: 69 (27.1%) CPI 4: 26 (10.2%)	OHIP-14 Total scores Age, gender, family income, years of education; BMI, diabetes mellitus, Kt/v, DMFT, CPI, QVPI, GI, oral health knowledge, oral health attitude, dental brushing, dental flossing, last dentist visit, smoking, general health-related quality of life	C: Periodontal clinical variables and OHIP-14 scores
Hajian-Tilaki *et al. *2014 [28] Iran	Cross-sectional Hemodialysis patients: Hemodialysis Center Control: None	n:145 53.1% Male 58.17±17.76	Plaque index, Periodontal disease index and simplified oral hygiene index (OHI-S) NR	OHIP-14 (ADD and SC) GOHAI (ADD and SC) Total scores (ADD) Binary OHRQoL outcome (SC) NR	C: PDI and OHIP-14 or GOHAI scores
Guzeldemir *et al.* 2009 [29] Turkey	Cross-sectional Hemodialysis patients: University Hemodialysis Center Control: None	n:47 51.1% Male 46.38±15.10	Plaque index, Gingival index, PPD and BOP NR	OHIP-14 (ADD and SC) GOHAI (ADD and SC) Total scores (ADD) Binary OHRQoL outcome (SC) NR	C: Periodontal clinical variables and OHIP-14 or GOHAI scores
**Author,** **Year** **Country**		**OHRQoL scores** *mean±SD*	**Correlation between periodontal clinical variables and OHRQoL**	**Impact of periodontal conditions on OHRQoL ***Relative risk: RR: (IC 95%)* **Domains affected**	
Oliveira *et al.* 2020 [23] Brazil		Total score: OHIP-14:7.25±7.69 Periodontitis: Severe: 8.50±8.46 Mild/moderate: 6.20±6.98 No: 4.75±5.69	NR	Periodontitis: Severe: RR: 1.77 (1.36-2.30) Mild/moderate: RR: 1.49 (1.16-1.91) Visible plaque index >15%: RR:1.31 (1.11-1.54) Domains: Physical pain; Physical disability; Psychological disability; Psychological discomfort	
Kahar *et al*. 2019 [27] United States		Total score: median (IQR) OHIP-14: 64.0 (54.8-68.0) GOHAI: 52.0 (39.8-56.3)	NR	No associations between CPI and any of the 14 OHIP items or the 12 GOHAI items.	
Schmalz *et al.* 2018 [30] Germany		Total score: mean (median, 25-75 percentile) OHIP-14: 4.17 (2.0;0-5.0) PPD≥4mm: 5.82 (2.0, 0-8.5) PPD<4mm: 3.15 (1.0, 0-3.0) *p=*0.030	Spearman Correlation PPD: r=0.164; *p=*0.03	NR	
Schmalz *et al.* 2016 [22] Germany		Hemodialysis: Total score: OHIP-14: 2.46±4.68 Periodontitis: Moderate/severe: 2.52±4.75 No/mild: 0.67±1.15 *p=*0.482 Kidney transplantations: Total score: OHIP-14: 2.54±3.68 Periodontitis: Moderate/severe: 2.65±3.85 No/mild: 1.75±2.06 *p=*0.847 Healthy: Total score: OHIP-14:1.52±2.71 Periodontitis: Moderate/severe: 1.79±2.92 No/mild: 0.47±1.26 *p=*0.023	NR	NR	
Pakpour *et al.* 2015 [26] Iran		Total score: OHIP-14 Hemodialysis: 19.10±10.21 Healthy: 9.66±5.24	Linear Regression: CPI: β=0.136; *p<*0.05 QVPI: β=0.088; *p<*0.05 GI: β=0.255; *p<*0.05	NR	
Hajian-Tilaki *et al.* 2014 [28] Iran		Total score: OHIP-14-ADD: 31.32±12.53 OHIP-14 SC: 1.92±3.36 GOHAI-ADD: 29.07±8.50 GOHAI-SC: 2.54±2.48 OHIP-14-ADD: *p=*0.040 OHI-S Good: 20.86±6.97 OHIP-14-ADD: *p=*0.040 OHI-S Good: 20.86±6.97 OHI-S Fair: 30.44±8.77 OHI-S Poor: 32.38±12.40 OHIP-14-SC: *p=*0.130 OHI-S Good: 0.0±0.0 OHI-S Fair: 1.0±2.1 OHI-S Poor: 2.0±3.4 GOHAI-ADD: *p=*0.010 OHI-S Good: 22.00±4.47 OHI-S Fair: 28.52±5.79 OHI-S Poor: 31.01±8.62 GOHAI-SC: *p=*0.044 OHI-S Good: 1.0±0.8 OHI-S Fair: 2.0±1.7 OHI-S Poor: 3.0±2.6	Pearson correlation: OHIP-14-ADD: PI: r=0.374; *p=*0.001 PDI: r=0.40; *p=*0.001 OHIP-14-SC: PI: r=0.294; *p=*0.004 PDI: r=0.262; *p=*0.01 GOHAI-ADD: PI: r=0.408; *p=*0.001 PDI: r=0.449; *p=*0.001 GOHAI-SC: PI: r=0.345; *p=*0.001 PDI: r=0.343; *p=*0.001	NR	
Guzeldemir *et al.* 2009 [29] Turkey		Total score: OHIP-14: 19.40±7.74 GOHAI: 15.72±8.68	Pearson correlation: OHIP-14-ADD: PI: r=0.344; *p=*0.025 GI: r=0.125; *p=*0.430 PPD: r=0.093; *p=*0.559 BOP: r=0.161; *p=*0.308 OHIP-14-SC: PI: r=0.249; *p=*0.107 GI: r=0.026; *p=*0.868 PPD: r=0.055; *p=*0.726 BOP: r=0.099; *p=*0.528 GOHAI-ADD: PI: r=0.265; *p=*0.094 GI: r=0.318; *p=*0.160 PPD: r=0.015; *p=*0.926 BOP: r=0.122; *p=*0.447 GOHAI-SC: PI: r=0.212; *p=*0.172 GI: r=0.099; *p=*0.528 PPD: r=-0.011; *p=*0.945 BOP: r=0.117; *p=*0.457	NR	

ADD= Additive; BMI= Body Mass Index; BOP= Bleeding on Probing

**Table 2:** Table 2: Quality assessment of included studies was conducted based on the number of stars (points) according to the Newcastle-Ottawa scale adapted for cross-sectional studies (n=7).

**Study**	**Selection**	**Comparability**	**Exposure**	**Outcome**	Max 9 *	**Quality**
Oliveira *et al.* 2020 [23]	*	*	**	*	5/9	Moderate
Kahar *et al.* 2019 [27]	*	*	*--*	*	3/9	Low
Schmalz *et al.* 2018 [30]	**	*	*--*	--	3/9	Low
Schmalz *et al.* 2016 [22]	*	-	*	--	2/9	Low
Pakpour *et al*. 2015 [26]	*	*	*	**	5/9	Moderate
Hajian-Tilaki *et al.* 2014 [28]	*	--	--	**	3/9	Low
Guzeldemir *et al.* 2009 [29]	*--*	--	*--*	*	1/9	Low

**Table 3:** Table 3: Pooled correlation coefficient between periodontal clinical parameters and OHRQoL scores (FQ2).

**Periodontal parameter**	**Studies**	**Number of participants**	**Pooled correlation coefficient (r)**	**95% CI**	*p*	**I** ^2^
Periodontal Probing Depth (OHIP-14)	Schmalz *et al.* 2018 [30] Guzeldemir et al. 2009 [29]	237	0.145	0.016-0.273	*p=*0.028	0%
Plaque index (OHIP-14)	Hajian-Tilaki *et al.* 2014 [28] Guzeldemir et al. 2009 [29]	192	0.385	0.241-0.529	*p<*0.001	0%
Plaque Index (GOHAI)	Hajian-Tilaki *et al.* 2014 [28] Guzeldemir et al. 2009 [29]	192	0.395	0.251-0.539	*p<*0.001	0%

CI: confidence interval; I2: Heterogeneity index

**Figure 1:**
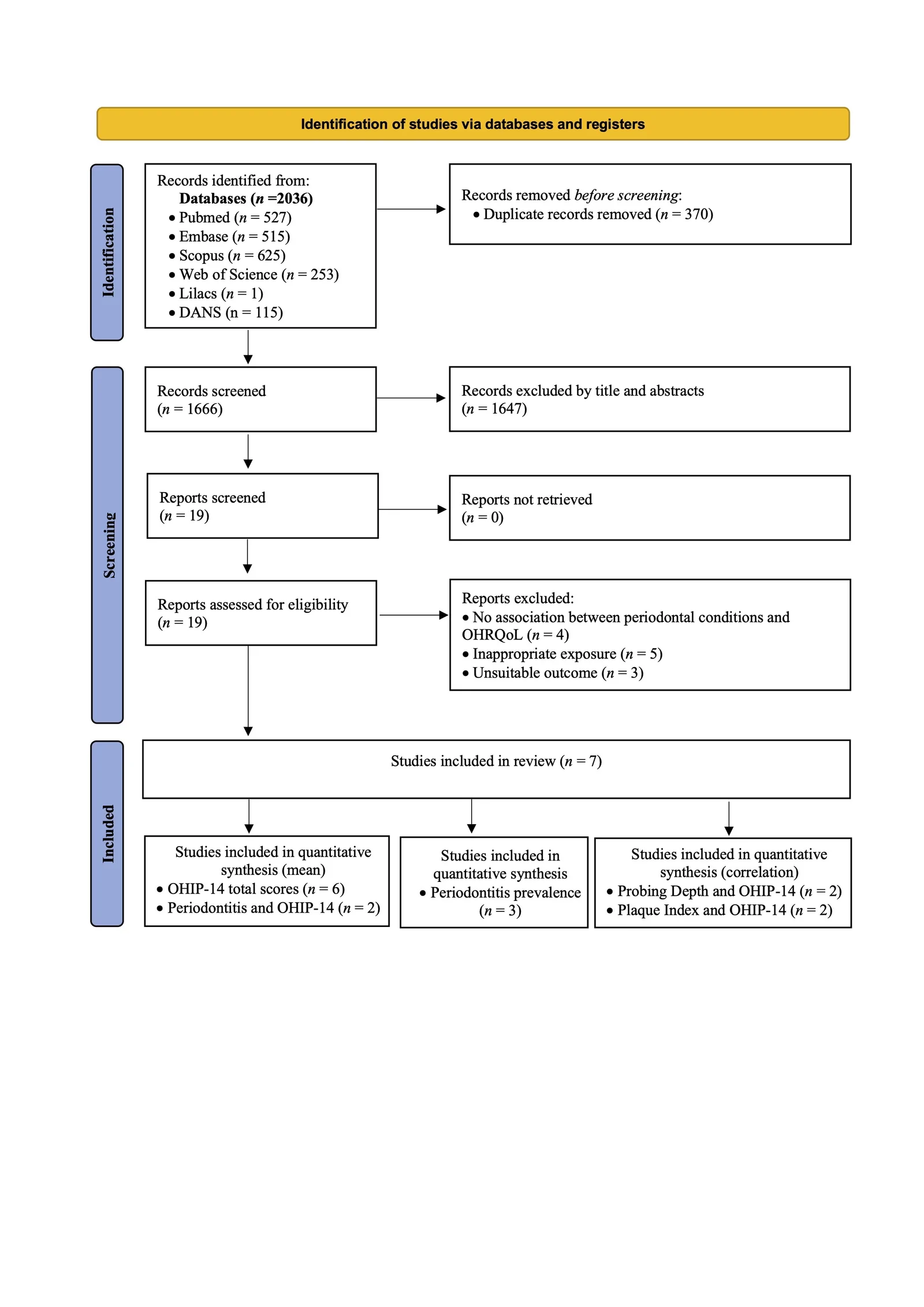
Fig. 1: PRISMA flowchart of the study selection process.

**Figure 2:**
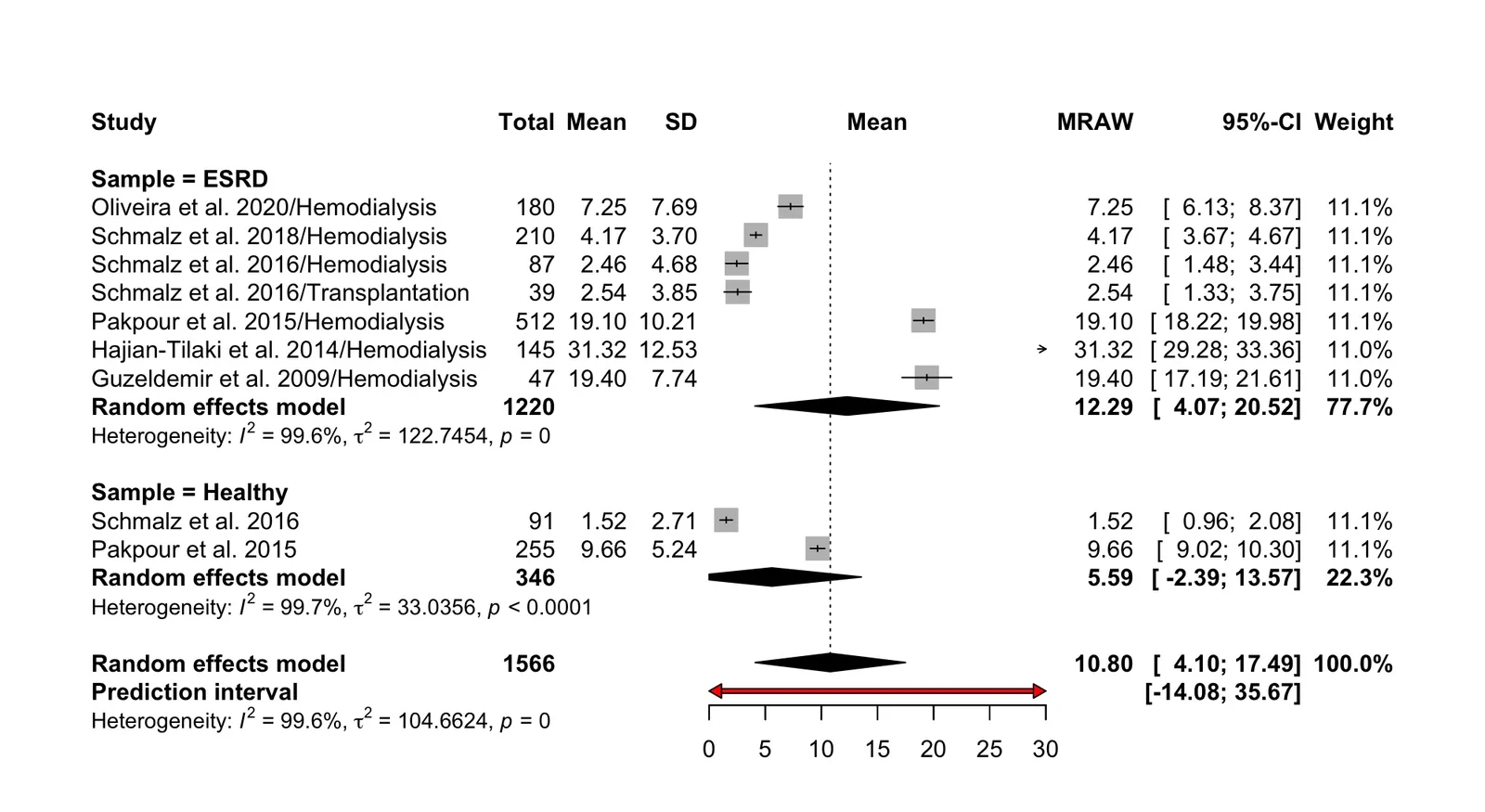
Fig. 2: Pooled estimates of OHRQoL in individuals with end-stage renal disease (ESRD) compared to systemically healthy controls (n=6 studies) (CI: confidence interval; I2: Heterogeneity index).

**Figure 3:**
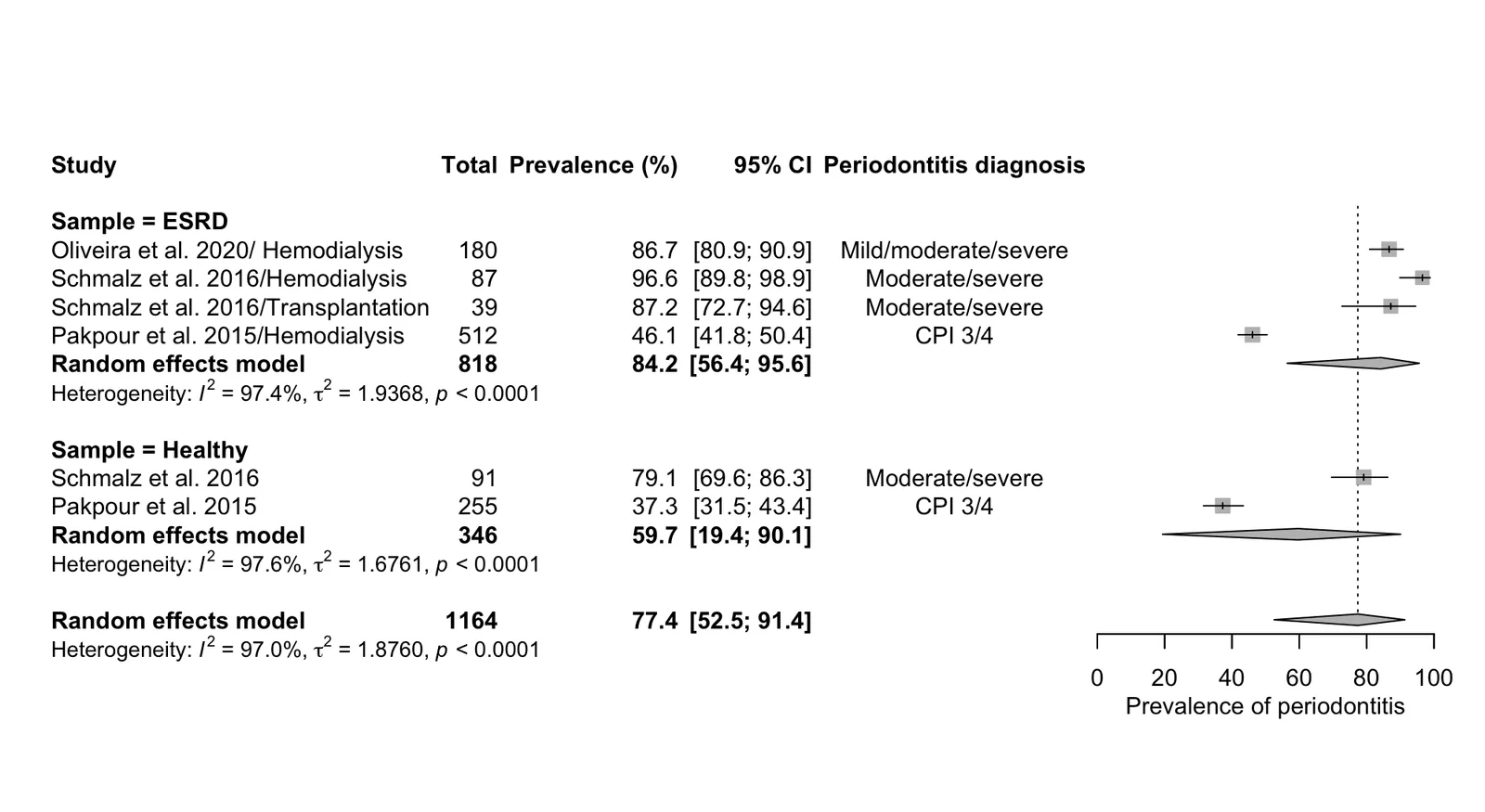
Fig. 3: Pooled estimates of periodontitis prevalence in individuals with end-stage renal disease (ESRD) compared to systemically healthy controls (n=3 studies) (CI: confidence interval; CPI: Community Periodontal Index; I2: Heterogeneity index).

**Figure 4:**
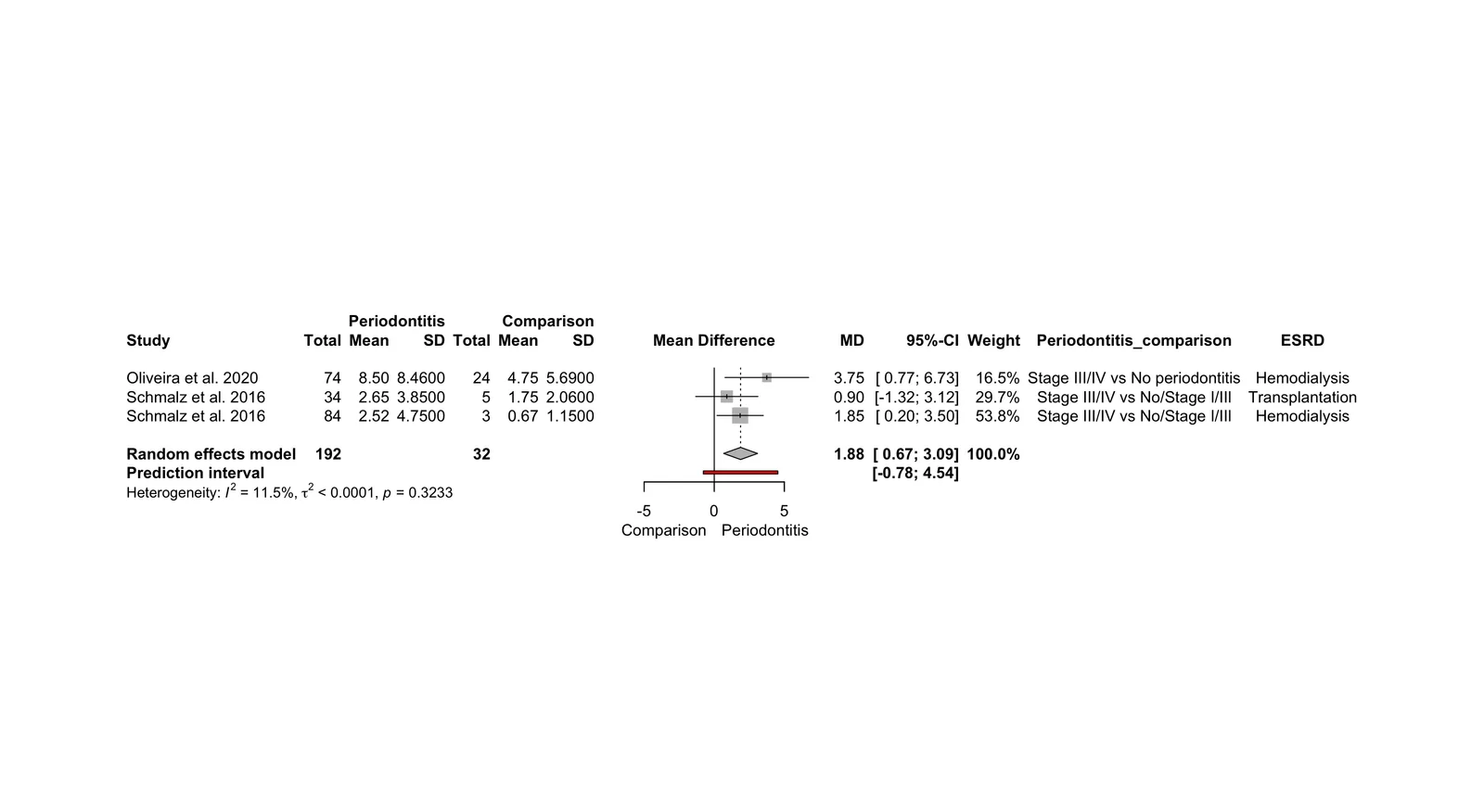
Fig. 4: Pooled estimates of mean differences (MD) in OHIP-14 scores among individuals with end-stage renal disease (ESRD), comparing stage III/IV periodontitis to those without periodontitis or with stage I/II periodontitis (n = 2 studies) (FQ1). (CI: confidence interval; I2: Heterogeneity index).

## Data Availability

None.

## References

[1] Kidney Disease: Improving Global Outcomes (KDIGO) CKD Work Group. KDIGO 2024 Clinical Practice Guideline for the Evaluation and Management of Chronic Kidney Disease. Kidney Int. 2024;105(4S): S117-S314. 10.1016/j.kint.2023.10.018 38490803

[2] Bello AK, Okpechi IG, Levin A, Ye F, Damster S, Arruebo S, Donner JA, Caskey FJ, Cho Y, Davids MR, Davison SN, Htay H, Jha V, Lalji R, Malik C, Nangaku M, See E, Sozio SM, Tonelli M, Wainstein M, Yeung EK, Johnson DW; ISN-GKHA Group. An update on the global disparities in kidney disease burden and care across world countries and regions. Lancet Glob Health. 2024;12(3): e382-e395. 10.1016/S2214-109X(23)00570-3 38365413

[3] Qing X, Jiang J, Yuan C, Xie K, Wang K (2024). Temporal trends in prevalence and disability of chronic kidney disease caused by specific etiologies: An analysis of the Global Burden of Disease Study 2019. J Nephrol.

[4] Luyckx VA, Tonelli M, Stanifer JW. The global burden of kidney disease and the sustainable development goals. Bull World Health Organ. 2018;96(6):414-422D. 10.2471/BLT.17.206441 PMC5996218 29904224

[5] Pagels AA, Söderkvist BK, Medin C, Hylander B, Heiwe S. Health-related quality of life in different stages of chronic kidney disease and at initiation of dialysis treatment.Health Qual Life Outcomes. 2012;10:71. 10.1186/1477-7525-10-71 PMC3511211 22710013

[6] Wyld MLR, Morton RL, Clayton P, Wong MG, Jardine M, Polkinghorne K, Chadban S (2019). The impact of progressive chronic kidney disease on health-related quality-of-life: A 12-year community cohort study. Qual Life Res.

[7] Tonetti MS, Greenwell H, Kornman KS. Staging and grading of periodontitis: Framework and proposal of a new classification and case definition. J Clin Periodontol. 2018;45 Suppl 20: S149-S161. 10.1111/jcpe.12945 29926495

[8] Nascimento GG, Alves-Costa S, Romandini M (2024). Burden of severe periodontitis and edentulism in 2021, with projections up to 2050: The Global Burden of Disease 2021 study. J Periodontal Res.

[9] Hajishengallis G (2022). Interconnection of periodontal disease and comorbidities: Evidence, mechanisms, and implications. Periodontol 2000.

[10] He I, Poirier B, Jensen E, Kaur S, Hedges J, Jesudason S, Jamieson L, Sethi S (2023). Demystifying the connection between periodontal disease and chronic kidney disease - An umbrella review. J Periodontal Res.

[11] Graziani F, Tsakos G (2020). Patient-based outcomes and quality of life. Periodontol 2000.

[12] Buset SL, Walter C, Friedmann A, Weiger R, Borgnakke WS, Zitzmann NU (2016). Are periodontal diseases really silent? A systematic review of their effect on quality of life. J Clin Periodontol.

[13] Ferreira MC, Dias-Pereira AC, Branco-de-Almeida LS, Martins CC, Paiva SM (2017). Impact of periodontal disease on quality of life: A systematic review. J Periodontal Res.

[14] Paśnik-Chwalik B, Konopka T (2020). Impact of periodontitis on the Oral Health Impact Profile: A systematic review and meta-analysis. Dent Med Probl.

[15] Agnese CCD, Schöffer C, Kantorski KZ, Zanatta FB, Susin C, Antoniazzi RP (2025). Periodontitis and Oral Health-Related Quality of Life: A Systematic Review and Meta-Analysis. J Clin Periodontol.

[16] Page MJ, McKenzie JE, Bossuyt PM, Boutron I, Hoffmann TC, Mulrow CD, Shamseer L, Tetzlaff JM, Moher D (2021). Updating guidance for reporting systematic reviews: Development of the PRISMA 2020 statement. J Clin Epidemiol.

[17] Wells GA, Shea BJ, O'Connell D, Peterson JE, Welch V, M Losos M, Tugwell P. The Newcastle-Ottawa Scale (NOS) for Assessing the Quality of Nonrandomised Studies in Meta-Analyses, 2011. http://www.ohri.ca/programs/clinical_ epidemiology/oxford.asp.

[18] Chambrone L, Guglielmetti MR, Pannuti CM, Chambrone LA (2011). Evidence grade associating periodontitis to preterm birth and/or low birth weight: I. A systematic review of prospective cohort studies. J Clin Periodontol.

[19] Romandini P, Marruganti C, Romandini WG, Sanz M, Grandini S, Romandini M (2024). Are periodontitis and dental caries associated? A systematic review with meta-analyses. J Clin Periodontol.

[20] Schünemann HJ, Brozek J, Guyatt G, Oxman A. Handbook for Grading the Quality of Evidence and the Strength of Recommendations Using the GRADE Approach, 2013. Hamilton, Canada McMaster University.

[21] Schünemann HJ, Vist GE, Higgins JPT. Chapter 15: Interpreting Results and Drawing Conclusions. In Cochrane Handbook for Systematic Reviews of Interventions Version 6.5 (updated August 2024), edited by Higgins JPT, Thomas J, Chandler J, Cumpston M, Li T, Page MJ, et al, (Chichester, UK: Cochrane).

[22] Schmalz G, Kollmar O, Vasko R, Müller GA, Haak R, Ziebolz D (2016). Oral health-related quality of life in patients on chronic haemodialysis and after kidney transplantation. Oral Dis.

[23] Oliveira LM, Sari D, Schöffer C, Santi SS, Antoniazzi RP, Zanatta FB (2020). Periodontitis is associated with oral health-related quality of life in individuals with end-stage renal disease. J Clin Periodontol.

[24] Eke PI, Page RC, Wei L, Thornton-Evans G, Genco RJ (2012). Update of the case definitions for population-based surveillance of periodontitis. J Periodontol.

[25] Page RC, Eke PI (2007). Case definitions for use in population-based surveillance of periodontitis. J Periodontol.

[26] Pakpour AH, Kumar S, Fridlund B, Zimmer S (2015). A case-control study on oral health-related quality of life in kidney disease patients undergoing haemodialysis. Clin Oral Investig.

[27] Kahar P, Chapman C, Gupta J (2019). Assessment of the Effect of Oral Health on Quality of Life and Oral-Health Indicators among ESRD Patients in Southwest Florida: A Pilot Study. Int J Dent.

[28] Hajian-Tilaki A, Oliae F, Jenabian N, Hajian-Tilaki K, Motallebnejad M (2014). Oral health-related quality of life and periodontal and dental health status in Iranian hemodialysis patients. J Contemp Dent Pract.

[29] Guzeldemir E, Toygar HU, Tasdelen B, Torun D (2009). Oral health-related quality of life and periodontal health status in patients undergoing hemodialysis. J Am Dent Assoc.

[30] Schmalz G, Dietl M, Vasko R, Müller GA, Rothermund L, Keller F, Ziebolz D, Rasche FM (2018). Dialysis vintage time has the strongest correlation to psychosocial pattern of oral health-related quality of life - a multicentre cross-sectional study. Med Oral Patol Oral Cir Bucal.

[31] Stenvinkel P, Larsson TE (2013). Chronic kidney disease: A clinical model of premature aging. Am J Kidney Dis.

[32] Mühlberg S, Jäger J, Krohn-Grimberghe B, Patschan S, Mausberg RF, Schmalz G, Haak R, Ziebolz D (2017). Oral health-related quality of life depending on oral health in patients with rheumatoid arthritis. Clin Oral Investig.

[33] Schmalz G, Meisel A, Kollmar O, Kauffels A, Slotta JE, Kottmann T, Haak R, Ziebolz D (2018). Oral health-related quality of life depending on dental and periodontal health in different patients before and after liver transplantation. Clin Oral Investig.

[34] Angst PDM, Dutra DAM, Manso IS, Moreira CHC, Kantorski KZ (2020). Association between oral health-related quality of life and periodontal status in patients with leukemia. Int Dent J.

[35] Schmalz G, Patschan S, Patschan D, Ziebolz D (2020). Oral health-related quality of life in adult patients with end-stage kidney diseases undergoing renal replacement therapy - a systematic review. BMC Nephrol.

[36] Patel RR, Richards PS, Inglehart MR (2008). Periodontal health, quality of life, and smiling patterns--an exploration. J Periodontol.

[37] Chapple IL, Van der Weijden F, Doerfer C, Herrera D, Shapira L, Polak D, Madianos P, Louropoulou A, Machtei E, Donos N, Greenwell H, Van Winkelhoff AJ, Eren Kuru B, Arweiler N, Teughels W, Aimetti M, Molina A, Montero E, Graziani F. Primary prevention of periodontitis: Managing gingivitis. J Clin Periodontol. 2015;42 Suppl 16: S71-6. 10.1111/jcpe.12366 25639826

[38] Riva F, Seoane M, Reichenheim ME, Tsakos G, Celeste RK (2022). Adult oral health-related quality of life instruments: A systematic review. Community Dent Oral Epidemiol.

[39] Slade GD (1997). Derivation and validation of a short-form oral health impact profile. Community Dent Oral Epidemiol.

