# Supplementary material for: Influence of periodontal conditions on quality of life in patients with chronic kidney disease: A systematic review and meta-analysis
Source: Med Oral Patol Oral Cir Bucal. 2026 Apr 19;31(5):e639–50. doi: 10.4317/medoral.28048 (PMC13632525; doi:10.4317/medoral.28048)
Supplement: Supplementary file 2 [file medoral-31-05-e639-s002.pdf]

Appendix S2. Search strategies used in the respective electronic databases

Database	Search strategy	Hits
**Pubmed/MEDLINE**<br><br>**(https://www.ncbi.nlm.nih.gov/pubmed)**	((("renal insufficiency, chronic"[MeSH Terms])) OR (Chronic Renal Insufficiencies)) OR (Renal Insufficiencies, Chronic)) OR (Chronic Renal Insufficiency)) OR (Kidney Insufficiency, Chronic)) OR (Chronic Kidney Insufficiency)) OR (Chronic Kidney Insufficiencies)) OR (Kidney Insufficiencies, Chronic)) OR (Chronic Kidney Diseases)) OR (Chronic Kidney Disease)) OR (Diseases, Chronic Kidney)) OR (Kidney Disease, Chronic)) OR (Kidney Diseases, Chronic)) OR (Chronic Renal Diseases)) OR (Chronic Renal Disease)) OR (Disease, Chronic Renal)) OR (Diseases, Chronic Renal)) OR (Renal Disease, Chronic)) OR (Renal Diseases, Chronic)) OR ("kidney failure, chronic"[MeSH Terms])) OR (End-Stage Kidney Disease)) OR (Disease, End-Stage Kidney)) OR (End Stage Kidney Disease)) OR (Kidney Disease, End-Stage)) OR (Chronic Kidney Failure)) OR (End-Stage Renal Disease)) OR (Disease, End-Stage Renal)) OR (End Stage Renal Disease)) OR (Renal Disease, End-Stage)) OR (Renal Disease, End Stage)) OR (Renal Failure, End-Stage)) OR (End-Stage Renal Failure)) OR (Renal Failure, End Stage)) OR (Renal Failure, Chronic)) OR (Chronic Renal Failure)) OR (ESRD)) OR ("renal dialysis"[MeSH Terms])) OR (Dialyses, Renal)) OR (Renal Dialyses)) OR (Dialysis, Renal)) OR (Hemodialysis)) OR (Hemodialyses)) OR (Dialysis, Extracorporeal)) OR (Dialyses, Extracorporeal)) OR (Extracorporeal Dialyses)) OR (Extracorporeal Dialysis)) OR ("glomerular filtration rate"[MeSH Terms])) OR (Filtration Rate, Glomerular)) OR (Filtration Rates, Glomerular)) OR (Glomerular Filtration Rates)) OR (Rate, Glomerular Filtration)) OR (Rates, Glomerular Filtration)) AND ((("periodontal diseases"[MeSH Terms]) OR (Disease, Periodontal)) OR (Diseases, Periodontal)) OR (Periodontal Disease)) OR (Parodontosis)) OR (Parodontoses)) OR (Pyorrhea Alveolaris)) OR ("periodontitis"[MeSH Terms])) OR ("chronic periodontitis"[MeSH Terms])) OR ("aggressive periodontitis"[MeSH Terms])) OR (Periodontitides)) OR (Pericementitis)) OR (Pericementitides)) OR (Chronic Periodontitides)) OR (Periodontitides, Chronic)) OR (Periodontitis, Chronic)) OR (Adult Periodontitis)) OR (Adult Periodontitides)) OR (Periodontitides, Adult)) OR (Periodontitis, Adult)) OR (Periodontitis, Aggressive)) OR (Periodontitis, Prepubertal)) OR (Periodontitis, Circumpubertal)) OR (Circumpubertal Periodontitis)) OR (Prepubertal Periodontitis)) OR (Early-Onset Periodontitis)) OR (Early Onset Periodontitis)) OR (Periodontitides, Early-Onset)) OR (Periodontitis, Early-Onset)) OR (Juvenile Periodontitis)) OR (Periodontitis, Juvenile)) OR (Juvenile Periodontitides)) OR (Periodontitides, Juvenile)) OR (Periodontosis)) OR (Periodontoses)) OR (Periodontitis, Aggressive, 1)) OR (Periodontal Pocket)) OR ("dental caries"[MeSH Terms])) OR (Dental Decay)) OR (Caries, Dental)) OR (Decay, Dental)) OR (Carious Dentin)) OR (Carious Dentins)) OR (Dentin, Carious)) OR (Dentins, Carious)) OR ("tooth loss"[MeSH Terms])) OR (Loss, Tooth)) OR (Teeth Lost)) OR ("xerostomia"[MeSH Terms])) OR ("oral health"[MeSH Terms])) OR ("mouth diseases"[MeSH Terms])) OR ("pathology, oral"[MeSH Terms])) OR (Xerostomias)) OR (Hyposalivation)) OR (Hyposalivations)) OR (Mouth Dryness)) OR (Dryness, Mouth)) OR (Salivary Flow Rate)) OR (Salivary Flow)) OR (Health, Oral)) OR (Disease, Mouth)) OR (Diseases, Mouth)) OR (Mouth Disease)) OR (Oral Conditions)) OR (Oral and Maxillofacial Pathology)) OR (Oral Pathology)) OR (Pathology, Maxillofacial)) OR (Maxillofacial Pathology))) AND ((((((("quality of life"[MeSH Terms]) OR (Life Quality)) OR (Health-Related Quality Of Life)) OR (Health Related Quality Of Life)) OR (HRQOL)) OR (Life Qualities)) OR (Oral Health-Related Quality Of Life))	527
**Embase**<br><br>**(https://www.embase.com)**	('chronic kidney failure'/exp OR 'chronic kidney disease' OR 'chronic kidney disorder' OR 'chronic kidney failure' OR 'chronic kidney insufficiency' OR 'chronic nephropathy' OR 'chronic renal disease' OR 'chronic renal insufficiency' OR 'kidney chronic failure' OR 'kidney disease, chronic' OR 'kidney failure, chronic' OR 'kidney function, chronic disease' OR 'renal insufficiency, chronic' OR 'hemodialysis'/exp OR 'blood dialysis' OR 'chronic haemodialysis' OR 'chronic hemodialysis' OR 'chronic intermittent haemodialysis' OR 'chronic intermittent hemodialysis' OR 'dialysis center' OR 'dialysis, blood' OR 'extracorporeal blood cleansing' OR 'extracorporeal dialysis' OR 'haemodialysis' OR 'haemodialysis center' OR 'haemodialysis centre' OR 'haemodialysis department' OR 'haemodialysis unit' OR 'haemodialysis units, hospital' OR 'hemodialyse' OR 'hemodialysis' OR 'hemodialysis center' OR 'hemodialysis department' OR 'hemodialysis unit' OR 'hemodialysis units, hospital' OR 'hemorenodialysis' OR 'hemotrialysate' OR 'hospital haemodialysis units' OR 'hospital hemodialysis units' OR 'intermittent chronic haemodialysis' OR 'intermittent chronic hemodialysis' OR 'intermittent haemodialysis' OR 'intermittent hemodialysis' OR 'renal dialysis' OR 'glomerulus filtration rate'/exp OR 'gfr (glomerulus filtration rate)' OR 'glomerular filtration rate' OR 'glomerulofiltration rate' OR 'glomerulus filtration rate' OR 'kidney gfr' OR 'kidney glomerulus filtration rate' OR 'end stage renal disease'/exp OR 'esrd' OR 'end stage kidney disease' OR 'end stage	515

	kidney failure' OR 'end stage renal disease' OR 'end stage renal dysfunction' OR 'end stage renal failure' OR 'end stage renal impairment' OR 'end stage renal insufficiency' OR 'end-stage kidney disease' OR 'end-stage kidney failure' OR 'end-stage renal disease' OR 'stage 5 kidney disease' OR 'stage 5 renal disease') AND ('periodontal disease'/exp OR 'dental loss' OR 'dental migration' OR 'dental mobility' OR 'furcation defects' OR 'mesial movement of teeth' OR 'paradontal disease' OR 'paradontopathy' OR 'paraodontopathy' OR 'parodentopathy' OR 'parodontal disease' OR 'parodontium disease' OR 'parodontive tissue disease' OR 'peridontal disease' OR 'peridontal tissue disease' OR 'peridontium disease' OR 'periodontal atrophy' OR 'periodontal attachment loss' OR 'periodontal disease' OR 'periodontal diseases' OR 'periodontal infection' OR 'periodontium disease' OR 'periodontopathy' OR 'tooth loss' OR 'tooth migration' OR 'tooth mobility' OR 'tooth movement' OR 'periodontitis'/exp OR 'paradontitis' OR 'parodontitis' OR 'peridontitis' OR 'periodontitis' OR 'chronic periodontitis'/exp OR 'adult periodontitis' OR 'chronic periodontitis' OR 'periodontitis chronica' OR 'aggressive periodontitis'/exp OR 'aggressive periodontitis' OR 'early onset periodontitis' OR 'juvenile periodontitis' OR 'periodontitis, juvenile' OR 'prepubertal periodontitis' OR 'periodontal pocket'/exp OR 'periodontal pocket' OR 'periodontal pocket depth'/exp OR 'depth of periodontal pocket' OR 'periodontal pocket depth' OR 'periodontal probe depth' OR 'periodontal probing depth' OR 'pocket depth (periodontal)' OR 'pocket probing depth' OR 'probing depth (periodontal)' OR 'probing pocket depth' OR 'dental caries'/exp OR 'caries' OR 'caries, dental' OR 'cariogenesis' OR 'carious dentine' OR 'carious teeth' OR 'dental caries' OR 'dental caries susceptibility' OR 'dental decay' OR 'dental fissure' OR 'dental fissures' OR 'fissure, tooth' OR 'root caries' OR 'tooth caries' OR 'tooth decay' OR 'tooth fissure' OR 'xerostomia'/exp OR 'dry mouth' OR 'mouth dryness' OR 'oral dryness' OR 'xerostomia' OR 'xerostomy' OR 'zerostomiasis' OR 'salivation'/exp OR 'saliva flow' OR 'saliva release' OR 'saliva secretion' OR 'salivary flow' OR 'salivary gland secretion' OR 'salivary secretion' OR 'salivation' OR 'mouth disease'/exp OR 'diagnosis, oral' OR 'mouth abnormalities' OR 'mouth disease' OR 'mouth diseases' OR 'mouth pathology' OR 'oral diagnosis' OR 'oral disease' OR 'oral manifestations' OR 'oral pathology' OR 'stomatognathic diseases' OR 'oral mucosal disease'/exp OR 'mouth mucosa disease' OR 'oral mucosa disease' OR 'oral mucosa diseases' OR 'oral mucosa disorder' OR 'oral mucosa disorders' OR 'oral mucosa lesion' OR 'oral mucosa lesions' OR 'oral mucosal conditions' OR 'oral mucosal disease' OR 'oral mucosal diseases' OR 'oral mucosal disorder' OR 'oral mucosal disorders' OR 'oral mucosal lesion' OR 'oral mucosal lesions' OR 'oral mucosal pathology') AND ('quality of life'/exp OR 'hrql' OR 'health related quality of life' OR 'life quality' OR 'quality of life' OR 'life qualities' OR 'oral health-related quality of life')	
**Scopus**<br>**(https://www.scopus.com)**	"Renal Insufficiency, Chronic" OR "Kidney Failure, Chronic" OR "Renal Dialysis" OR "Glomerular Filtration Rate " OR "ESRD" AND "Periodontal Diseases" OR "Disease, Periodontal" OR "Diseases, Periodontal" OR "Periodontal Disease" OR "Periodontitis" OR "Chronic Periodontitis" OR "Aggressive Periodontitis" OR "Dental Caries" OR "Dental Decay" OR "tooth loss" OR "xerostomia" OR "Hyposalivation" OR "Mouth Dryness" AND "Quality of Life" OR "Oral Health-Related Quality Of Life"	625
**Web of Science**<br>**(https://login.webofknowledge.com)**	**Renal Insufficiency, Chronic OR Kidney Failure, Chronic OR Renal Dialysis OR Glomerular Filtration Rate (All fields) and Periodontal Diseases OR Chronic Periodontitis OR Aggressive Periodontitis OR Dental Caries OR Tooth Loss OR Xerostomia OR Oral Health OR Mouth Diseases (All fields) and (quality of life OR Life Quality OR Health-Related Quality Of Life OR Health Related Quality Of Life OR HRQOL OR Life Qualities) (All fields)**	253
**Lilacs**<br>**(lilacs.bvsalud.org)**	(renal insufficiency, chronic) OR (Chronic Renal Insufficiencies) OR (Renal Insufficiencies, Chronic) OR (Chronic Renal Insufficiency) OR (Kidney Insufficiency, Chronic) OR (Chronic Kidney Insufficiency) OR (Chronic Kidney Insufficiencies) OR (Kidney Insufficiencies, Chronic) OR (Chronic Kidney Diseases) OR (Chronic Kidney Disease) OR (Diseases, Chronic Kidney) OR (Kidney Disease, Chronic) OR (Kidney Diseases, Chronic) OR (Chronic Renal Diseases) OR (Chronic Renal Disease) OR (Disease, Chronic Renal) OR (Diseases, Chronic Renal) OR (Renal Disease, Chronic) OR (Renal Diseases, Chronic) OR (kidney failure, chronic) OR (End-Stage Kidney Disease) OR (Disease, End-Stage Kidney) OR (End Stage Kidney Disease) OR (Kidney Disease, End-Stage) OR (Chronic Kidney Failure) OR (End-Stage Renal Disease) OR (Disease, End-Stage Renal) OR (End Stage Renal Disease) OR (Renal Disease, End-Stage) OR (Renal Disease, End Stage) OR (Renal Failure, End-Stage) OR (End-Stage Renal Failure) OR (Renal Failure, End Stage) OR (Renal Failure, Chronic) OR (Chronic Renal Failure) OR (ESRD) OR (renal dialysis) OR (Dialyses, Renal) OR (Renal Dialyses) OR (Dialysis, Renal) OR (Hemodialysis) OR (Hemodialyses) OR (Dialysis, Extracorporeal) OR (Dialyses, Extracorporeal) OR (Extracorporeal Dialyses) OR (Extracorporeal Dialysis) OR (glomerular filtration rate) OR (Filtration Rate, Glomerular) OR (Filtration Rates, Glomerular) OR (Glomerular Filtration Rates) OR (Rate, Glomerular Filtration) OR	1

	(Rates, Glomerular Filtration) [Words] [Words] and (periodontal diseases) OR (Disease, Periodontal) OR (Diseases, Periodontal) OR (Periodontal Disease) OR (Parodontosis) OR (Parodontoses) OR (Pyorrhea Alveolaris) OR (periodontitis) OR (chronic periodontitis) OR (aggressive periodontitis) OR (Periodontitides) OR (Pericementitis) OR (Pericementitides) OR (Chronic Periodontitides) OR (Periodontitides, Chronic) OR (Periodontitis, Chronic) OR (Adult Periodontitis) OR (Adult Periodontitides) OR (Periodontitides, Adult) OR (Periodontitis, Adult) OR (Periodontitis, Aggressive) OR (Periodontitis, Prepubertal) OR(Periodontitis, Circumpubertal) OR (Circumpubertal Periodontitis) OR (Prepubertal Periodontitis) OR (Early-Onset Periodontitis) OR (Early Onset Periodontitis) OR (Early-Onset Periodontitides) OR (Periodontitides, Early-Onset) OR (Periodontitis, Early-Onset) OR (Juvenile Periodontitis) OR (Periodontitis, Juvenile) OR (Juvenile Periodontitides) OR (Periodontitides, Juvenile) OR (Periodontosis) OR (Periodontoses) OR (Periodontitis, Aggressive, 1) OR (Periodontal Pocket) OR (dental caries) OR (Dental Decay) OR (Caries, Dental) OR (Decay, Dental) OR (Carious Dentin) OR (Carious Dentins) OR (Dentin, Carious) OR (Dentins, Carious) OR (tooth loss) OR (Loss, Tooth) OR (Teeth Lost) OR (xerostomia) OR (Xerostomias) OR (Hyposalivation) OR (Hyposalivations) OR (Mouth Dryness) OR (Dryness, Mouth) OR (Salivary Flow Rate) OR (Salivary Flow) OR (oral health) OR (Health, Oral) OR (mouth diseases) OR (Disease, Mouth) OR (Diseases, Mouth) OR (Mouth Disease) OR (Oral Conditions) OR (pathology, oral) OR (Oral and Maxillofacial Pathology) OR (Oral Pathology) OR (Pathology, Maxillofacial) OR (Maxillofacial Pathology) [Words] [Words] and (quality of life) OR (Life Quality) OR (Health-Related Quality Of Life) OR (Health Related Quality Of Life) OR (HRQOL) OR (Life Qualities) [Words]	
**DANS (https://easy.dans.knaw.nl/ui/datasets/id/easydataset:200362/tab/)**	(Periodontal Diseases OR Chronic Periodontitis OR Aggressive Periodontitis OR Dental Caries OR Tooth Loss OR Xerostomia OR Oral Health OR Mouth Diseases and quality of life OR Life Quality OR Health-Related Quality of Life OR Health Related Quality Of Life OR HRQOL OR Life Qualities)	115
**Total**		2036
