# Supplementary material for: Influence of periodontal conditions on quality of life in patients with chronic kidney disease: A systematic review and meta-analysis
Source: Med Oral Patol Oral Cir Bucal. 2026 Apr 19;31(5):e639–50. doi: 10.4317/medoral.28048 (PMC13632525; doi:10.4317/medoral.28048)
Supplement: Supplementary file 3 [file medoral-31-05-e639-s003.pdf]

Appendix S3. Studies excluded after full text evaluation and reasons for exclusion.

Reason for Exclusion	Publication
No association between periodontal conditions and OHRQoL (n=4)	(Molania et al. 2023; Kahar et al., 2021; Oduncuoğlu et al., 2020; Camacho-Alonso et al., 2018)
Inappropriate exposure (n=5)	(Kwon et.al., 2024; Tabesh et al., 2022; Purisinsith et al., 2022; Lira e Silva et al., 2017; Andrade et al., 2017)
Unsuitable outcome (n=3)	(Joshi et al., 2024; Acosta-Ochoa et al., 2023; Ruokonen et al 2019)

REFERENCES (EXCLUDED STUDIES)

1. Acosta-Ochoa, I.; Tello-Pellitero, M.; Coca, A.; Rollán, M.J.; Sanz, S.; Ardura, P.; Moscoso, C.L.M.; Martinez, M.; Campuzano, K.P.C.; Mendiluce, A. Oral Manifestations, Periodontal Disease, and Need for Dental Treatment in Our Hemodialysis Unit. Journal of the American Society of Nephrology. 2023;34(0), 1077.
2. Andrade, A., Amorim, A., Queiroz, S., Gordón-Núñez, M., Freitas, R., & Galvão, H. Comparison of Oral Health Status and the Quality of Life in Haemodialysis Patients with Less and More than Four Years of Treatment. Oral Health & Preventive Dentistry. 2017; 15(1), 57–64.
3. Camacho-Alonso, F., Cánovas-García, C., Martínez-Ortiz, C., De la Mano-Espinosa, T., Ortuño-Celdrán, T., Marcello-Godino, J. I., Sánchez-Siles, M. Oral status, quality of life, and anxiety and depression in hemodialysis patients and the effect of the duration of treatment by dialysis on these variables. Odontology. 2018,106(2), 194–201.
4. Joshi, N. P., Shrestha, A., Bhagat, T., Agrawal, S. K., & Chhetri, R. The Oral Health Condition of Patients on Hemodialysis at a Tertiary Healthcare Facility in Eastern Nepal. International Journal of Dentistry. 2024; 3776702.
5. Kahar, P., Chapman, C. A., Brahmbhatt, S., & Khanna, D. Assessment of the Effects of Occluding Pairs of Teeth on Oral Health-related Quality of Life among Dialysis Patients. The Journal of Contemporary Dental Practice. 2021; 22(2), 156–161.
6. Lira E Silva, J. A., Bernardino, Í. de M., da Silva, J. R. C., Lima, T. L. M. de A., Soares, R. de S. C., & d'Ávila, S. Quality of life related to oral health of patients undergoing hemodialysis and associated factors. Special Care in Dentistry : Official Publication of the American Association of Hospital Dentists, the Academy of Dentistry for the Handicapped, and the American Society for Geriatric Dentistry. 2017;37(5), 236–245.
7. Molania, T.; Aryana, M.; Espahbodi, F.; Mesgarani, A.; Mehdinejad, A.; Salehi, M. Relationship between Oral Health Related Quality of Life and Hyposalivation and Xerostomia in Patients with Chronic Renal Failure Undergoing Hemodialysis. Journal of Mazandaran University of Medical Sciences. 2023; 33(224), 67–76.
8. Oduncuoğlu, B. F., Alaaddinoğlu, E. E., Çolak, T., Akdur, A., & Haberal, M. Effects of Renal Transplantation and Hemodialysis on Patient's General Health Perception and Oral Health-Related Quality of Life: A Single-Center Cross-Sectional Study. Transplantation Proceedings. 2020;52(3), 785–792.
9. Purisinsith, S., Kanjanabuch, P., Phannajit, J., Kanjanabuch, T., Puapatanakul, P., Johnson, D. W., Cheawchanwattana, A. Oral Health-Related Quality of Life, A Proxy of Poor Outcomes in Patients on Peritoneal Dialysis. Kidney International Reports. 2022; 7(10), 2207–2218.

10. Ruokonen, H., Nylund, K., Meurman, J. H., Heikkinen, A. M., Furuholm, J., Sorsa, T.,Ortiz, F. Oral symptoms and oral health-related quality of life in patients with chronic kidney disease from predialysis to posttransplantation. Clinical Oral Investigations. 2019; 23(5), 2207–2213.
11. Tabesh, A., Sadat Abtahi, M., Narimany, R., & Sadat Abtahi, M. Oral health-related quality of life in chronic kidney disease patients. Dental Research Journal. 2022; 19, 73.
12. Kwon EY, Joo JY. Assessment of oral health-related quality of life and periodontal state in patients with end-stage renal disease: a comparison study using propensity score-matched analysis. Med Oral Patol Oral Cir Bucal. 2024;29 (6):e768-74.
