# Supplementary material for: Influence of periodontal conditions on quality of life in patients with chronic kidney disease: A systematic review and meta-analysis
Source: Med Oral Patol Oral Cir Bucal. 2026 Apr 19;31(5):e639–50. doi: 10.4317/medoral.28048 (PMC13632525; doi:10.4317/medoral.28048)
Supplement: Supplementary file 4 [file medoral-31-05-e639-s004.pdf]

Appendix S4. Newcastle-Ottawa Scale

Newcastle-Ottawa quality assessment scale adapted for cross-sectional studies

Note: A maximum of one star (*) is awarded for each numbered item and a maximum of two stars (**) are awarded for the items “Representativeness” and “Adjustment for confounding factors”.

SELECTION (max. 2*)

1) Representativeness of the study sample:
a) Truly representative of the average in the target population (all subjects or random sampling). **
b) Somewhat representative of the average in the target population (non-random sampling). *
c) Selected group of users.
d) No description of the sampling strategy.

COMPARABILITY (max. 1*)
1) Comparison group:
a) Comparison group including only individuals without periodontitis *
b) Comparison group composed of individuals with periodontal health, gingivitis and stage I-II periodontitis or equivalent in the same group.

EXPOSURE (max. 2*)
1) Calibration for exposure (periodontal conditions):
a) Calibrated examiner (description of training/calibration procedure) *
b) Non-calibrated examiner (absence of calibration or report of calibration, but with no description of procedure).
2) Assessment of periodontal condition:
a) Adequate (diagnosis based on full mouth probing measurements, i.e., PPD and/or CAL) *
b) Inadequate (partial mouth recording or use of indexes with questionable value in describing the true periodontal status such as CPI)
d) Unclear (methods were not clear or not reported)

OUTCOME (max. 4*)
1) Evaluation of outcome (oral health-related quality of life (OHRQoL):
a) Blind evaluation (Assessment of quality of life with a self-administered instrument, administered by other researcher or before the clinical periodontal examination) *
b) Not blinded
2) Adjustments for confounding factors:
a) Adjustment for oral clinical confounders (xerostomia, dental caries, or tooth loss) **
b) Adjustment for other confounding factors *
c) None
3) Response rate:
a) All subjects selected for the study consented to be enrolled *
b) Subjects lost are unlikely to introduce bias - small number lost (response rate > 70%), or description provided of those lost) *
c) Response rate < 70 % and no description of those lost.
d) No statement
